# Changes in Mental Health of Women Undergoing Assisted Reproductive Technology Treatment During the COVID-19 Pandemic Outbreak in Xi'an, China

**DOI:** 10.3389/fpubh.2021.645421

**Published:** 2021-05-25

**Authors:** Pengfei Qu, Doudou Zhao, Peng Jia, Shaonong Dang, Wenhao Shi, Min Wang, Juanzi Shi

**Affiliations:** ^1^Translational Medicine Center, Northwest Women's and Children's Hospital, Xi'an, China; ^2^Assisted Reproduction Center, Northwest Women's and Children's Hospital, Xi'an, China; ^3^Departments of Pediatrics and Neonatology, Children's Hospital of Fudan University, Shanghai, China; ^4^Department of Land Surveying and Geo-Informatics, The Hong Kong Polytechnic University, Hong Kong, China; ^5^International Institute of Spatial Lifecourse Epidemiology (ISLE), Hong Kong, China; ^6^Department of Epidemiology and Health Statistics, School of Public Health, Xi'an Jiaotong University Health Science Center, Xi'an, China

**Keywords:** mental health, assisted reproductive technology, COVID-19, cross-sectional study, women

## Abstract

**Objective:** To investigate the mental health of women undergoing assisted reproductive technology (ART) treatment during the novel coronavirus pneumonia (COVID-19) pandemic outbreak in Xi'an, China.

**Methods:** A repeated cross-sectional study was administered to women undergoing ART treatment during the outbreak period (599 women in February 2020) and the control period (892 women in May 2020) at the Northwest Women's and Children's Hospital, Xi'an, China.

**Results:** Both the ART-treated women surveyed during the outbreak period and those surveyed during the control period had high scores on the fear dimension (0.88, 0.51). The total scores for mental health among the participants during the control period were lower than those during the outbreak period (difference = −0.22; 95% CI = −0.25, −0.18). Lower scores were also seen during the control period, compared to those in the outbreak period, for depression (difference = −0.18; 95% CI = −0.23, −0.13), neurasthenia (difference = −0.31; 95% CI = −0.36, −0.25), fear (difference = −0.37; 95% CI = −0.43, −0.31), compulsion anxiety (difference = −0.13; 95% CI = −0.16, −0.09), and hypochondriasis (difference = −0.09; 95% CI = −0.12, −0.06).

**Conclusions:** During the COVID-19 global pandemic, the mental health of women undergoing ART treatment in Xi'an, China, was primarily manifested as fear. As the pandemic was brought under control, the mental health of ART-treated women improved. As evidenced by these results, the COVID-19 pandemic influences the mental health of women undergoing ART treatment, and clinicians should be aware of this for similar future situations.

## Introduction

A novel coronavirus pneumonia (COVID-19) outbreak occurred in Wuhan, Hubei, China, at the end of 2019 (1). Since February 2020, the COVID-19 pandemic has shown rapid global escalation, and the cumulative number of confirmed cases has exploded (2). On March 11, the World Health Organization (WHO) officially declared COVID-19 a pandemic. Difficulties in fighting this global public health issue have been increasing (3). At present, although the pandemic shows fluctuations in China, such as the recent local resurgences in Heilongjiang, Beijing, and Xinjiang, the overall situation has been under control.

However, this raging pandemic may have had a heavy impact on the mental health of people around the world. As in the cases of the outbreak of severe acute respiratory syndrome (SARS) in 2003 and Ebola in 2014, the public has generally felt fear and anxiety and has thus overreacted due to the uncertainty of the virus during the incubation period and its possible transmission by asymptomatic infections (4, 5). After its outbreak, different levels of strict control strategies have been undertaken to curb the pandemic, including a city closure in Wuhan and community-based prevention and control in other regions, which may have adverse socio-psychological effects on citizens (6). Social media platforms, such as Weibo, WeChat, and We-Media, have been delivering exhaustive information about the pandemic. Such an information overload may inconspicuously exert further psychological pressure on citizens, such as in the form of depression, anxiety, or post-traumatic stress disorder (7, 8), particularly among high-risk populations (e.g., survivors, front-line medical staff) (9).

Most of the current studies on the impact of COVID-19 or SARS on mental health have also shown that women are more likely to experience anxiety or depression and post-traumatic stress due to the pandemic (10–13). The increased difficulty in accessing antenatal care during the pandemic has led pregnant women to feel more concerned about the healthy development of their fetuses (14–16). With the development of reproductive medicine, an increasing number of infertile couples become pregnant through assisted reproductive technology (ART) [including *in vitro* fertilization (IVF) and intracytoplasmic sperm injection (ICSI)] around the world. However, undergoing ART treatment is psychologically and emotionally stressful for most patients and involves the possibility of perceived fear, distress, depression, or anxiety (17–20). Due to the complexity, long treatment cycle, and high costs associated with ART treatment, the mental health of women undergoing such treatment is vulnerable and necessitates closer attention. A cohort study in Taiwan prior to COVID-19 showed that ART-treated women's mental health status was worst in the first trimester and within 2 months following delivery (21). However, no relevant research has been conducted on the mental health of ART-treated women during the COVID-19 pandemic. To fill this gap, this study investigated changes in the mental health of ART-treated women during the COVID-19 pandemic in Xi'an, Shaanxi Province, China.

## Materials and Methods

### Study Design and Population

Using an online psychological questionnaire, we conducted a repeated cross-sectional study during the outbreak period (February 2020) and the control period (May 2020) at the Assisted Reproduction Center of Northwest Women's and Children's Hospital in Xi'an, Shaanxi Province in northwestern China. In February 2020, 608 ART-treated women were invited to participate in the study, and nine refused, leaving 599 complete questionnaires. In May 2020, 909 ART-treated women were invited, and 17 refused. Therefore, a total of 1,491 complete questionnaires, 599 in February and 892 in May, were included in the final analysis.

### Mental Health Assessment

The Psychological Questionnaire for Public Health Emergencies was used to assess the mental health of the ART-treated women recruited (22). There are 27 items in the questionnaire, which are divided into five dimensions: depression, neurasthenia, fear, compulsive anxiety, and hypochondria; each item corresponded to a specific question. For each item, the participant scored 0, 1, 2, or 3 based on the degree (no, mild, moderate, severe) and frequency (occasionally, sometimes, often, always) of the emotional reaction that occurred at the time of survey. The total score in each dimension was divided by the number of items in that dimension to calculate that dimension's score. The higher the score of a particular dimension, the more severe the emotional reaction in that dimension.

### Covariates

Sociodemographic characteristics were also collected in the study. These characteristics include the participants' age (<30, 30–34.99, ≥35 years old), educational level (junior high school and below, high school/secondary school, college, or University and above), occupation (professional and technical personnel, administrative staff, workers/service staff, other, or unemployed), residence (city, town, or rural), family monthly income level (<2,500 yuan, 2,500–4,999 yuan, 5,000–9,999 yuan, or ≥10,000 yuan), number of children in the family (0 or ≥1), and phase of ART treatment (before IVF treatment, first cycle of IVF, second cycle of IVF, or third and later cycle of IVF).

### Statistical Analysis

The participants' sociodemographic characteristics were summarized using counts and proportions for categorical variables. Chi-squared tests were performed to compare the categorical variables. Mental health scores were described using mean and standard deviation for continuous variables, and analysis of covariance as well as a Wilcoxon rank test or Kruskal-Wallis test were performed to compare continuous variables. The univariate and multivariate generalized linear model was used to analyze the difference in mental health scores between the two waves. Additionally, a generalized linear model was used for subgroup analysis. Statistical analyses were executed with the SAS software package (version 9.4, SAS Institute Inc., Cary, NC, USA). All *P*-values were two-sided with a significance level of <0.05.

## Results

### Sociodemographic Characteristics

Forty women participated in both participant groups. The majority of participants were between 30 and 35 years old (50.58% for the outbreak period and 46.08% for the control period). The educational level accounting for the largest proportion of participants was University and above (33.89, 31.84%), and the occupation accounting for the largest proportion was unemployed (35.89, 30.61%). Most participants resided in rural areas (54.92, 58.07%), the most common monthly household income level 2,055–4,999 yuan (37.73, 36.32%), and the majority of couples had no children (91.32, 88.79%). The most common phase of ART treatment was the second cycle of IVF in the outbreak period (37.06%), but in the control period, most participants had not yet begun IVF treatment (40.81%). There was a statistically significant difference in occupation and phase of ART treatment (*P* = 0.026; *P* < 0.001); the distributions of other sociodemographic characteristics between the two waves were well-balanced (Table 1).

**Table 1:** Baseline characteristics of the study subjects (%).

**Variables**	**Outbreak period (*n* = 599)**	**Control period (*n* = 892)**	**χ^2^ value**	***P*-value**
**Women's age (year)**, ***n*** **(%)**			
<30	170 (28.38)	294 (32.96)		
30–34.99	303 (50.58)	411 (46.08)	3.936	0.140
≥35	126 (20.04)	187 (20.96)		
**Education level**, ***n*** **(%)**			
Junior high school and below	130 (21.70)	199 (22.31)	0.706	0.872
High school/secondary school	112 (18.70)	170 (19.06)		
College	154 (25.71)	239 (26.79)		
University and above	203 (33.89)	284 (31.84)		
**Occupation**, ***n*** **(%)**			
Professional and technical	114 (19.03)	140 (15.70)		
personnel				
Administrative staff	65 (10.85)	125 (14.01)		
Workers/service staff	89 (14.86)	154 (17.26)	11.067	0.026
Other	116 (19.37)	200 (22.42)		
Unemployed	215 (35.89)	273 (30.61)		
**Residence**, ***n*** **(%)**			
City	163 (27.21)	241 (27.02)		
Town	107 (17.86)	133 (14.91)	2.571	0.277
Rural	329 (54.92)	518 (58.07)		
Family monthly income level (yuan^*^), ***n*** **(%)**			
<2,500	103 (17.20)	139 (15.58)	6.448	0.092
2,055–4,999	226 (37.73)	324 (36.32)		
5,000–9,999	173 (28.88)	238 (26.68)		
≥10,000	97 (16.19)	191 (21.41)		
**Number of family children**			
0	547 (91.32)	792 (88.79)	2.505	0.114
≥1	52 (8.68)	100 (11.21)		
**Phases of ART treatment**			
Before IVF treatment	164 (27.38)	364 (40.81)	195.102	<0.001
First cycle of IVF	100 (16.69)	338 (37.89)		
Second cycle of IVF	222 (37.06)	127 (14.24)		
Third and later cycle of IVF	113 (18.86)	63 (7.06)		

*IVF, in vitro fertilization*.

* *Exchange rate of USD to RMB 1–7*.

### The Psychological State of ART-treated Women

The ART-treated women surveyed during the outbreak period and those surveyed during the control period both had the highest scores for the fear dimension (0.88, 0.51), high scores for the neurasthenia dimension (0.60, 0.26) and depression dimension (0.41, 0.21), low scores for the compulsive-anxiety dimension (0.23, 0.09), and the lowest scores for the hypochondria dimension (0.14, 0.04). Moreover, we found that the fear dimension scores for different education levels, residence locations, and family monthly income levels were statistically different during the outbreak period (*P* < 0.001, *P* = 0.008, *P* = 0.003). Women who had a higher education level and higher family income and who lived in cities had higher scores for the fear dimension (see Table 2 for details).

**Table 2:** The psychological state of ART-treated women during the outbreak period and during the control period (Mean ± SD).

**Variables**	**Outbreak period**						**Control period**					
	**Depression score**	**Neurasthenia score**	**Fear score**	**Compulsive-anxiety score**	**Hypochondria score**	**Total score**	**Depression score**	**Neurasthenia score**	**Fear score**	**Compulsive-anxiety score**	**Hypochondria score**	**Total score**
Total	0.41 ± 0.52	0.60 ± 0.59	0.88 ± 0.55	0.23 ± 0.39	0.14 ± 0.34	0.47 ± 0.40	0.21 ± 0.35	0.26 ± 0.39	0.51 ± 0.45	0.09 ± 0.22	0.04 ± 0.17	0.24 ± 0.26
**Women's age (year)**											
<30	0.35 ± 0.48	0.58 ± 0.60	0.85 ± 0.53	0.20 ± 0.39	0.12 ± 0.31	0.44 ± 0.39	0.20 ± 0.36	0.26 ± 0.39	0.51 ± 0.46	0.09 ± 0.23	0.03 ± 0.13	0.23 ± 0.27
30–34.99	0.43 ± 0.52	0.59 ± 0.58	0.87 ± 0.57	0.24 ± 0.38	0.15 ± 0.37	0.48 ± 0.41	0.20 ± 0.34	0.24 ± 0.38	0.49 ± 0.44	0.09 ± 0.22	0.04 ± 0.19	0.23 ± 0.25
≥35	0.42 ± 0.55	0.63 ± 0.59	0.94 ± 0.53	0.24 ± 0.41	0.13 ± 0.33	0.50 ± 0.41	0.20 ± 0.36	0.31 ± 0.40	0.55 ± 0.46	0.10 ± 0.23	0.06 ± 0.17	0.27 ± 0.26
*P-*value	0.258	0.767	0.298	0.519	0.579	0.397	0.388	0.093	0.297	0.987	0.150^*^	0.166
**Education level**											
Junior high school and below	0.42 ± 0.55	0.59 ± 0.62	0.78 ± 0.59	0.25 ± 0.40	0.10 ± 0.25	0.46 ± 0.42	0.22 ± 0.38	0.27 ± 0.41	0.53 ± 0.53	0.12 ± 0.27	0.05 ± 0.19	0.26 ± 0.30
High school/secondary school	0.34 ± 0.52	0.51 ± 0.59	0.77 ± 0.60	0.22 ± 0.46	0.11 ± 0.33	0.41 ± 0.44	0.22 ± 0.37	0.23 ± 0.34	0.50 ± 0.46	0.09 ± 0.23	0.05 ± 0.17	0.24 ± 0.27
College	0.38 ± 0.49	0.60 ± 0.59	0.89 ± 0.50	0.20 ± 0.37	0.13 ± 0.31	0.45 ± 0.37	0.22 ± 0.36	0.25 ± 0.39	0.48 ± 0.43	0.10 ± 0.22	0.03 ± 0.16	0.24 ± 0.26
University and above	0.46 ± 0.51	0.63 ± 0.55	1.00 ± 0.52	0.25 ± 0.36	0.19 ± 0.42	0.53 ± 0.38	0.19 ± 0.31	0.28 ± 0.40	0.53 ± 0.40	0.08 ± 0.17	0.04 ± 0.16	0.24 ± 0.21
*P-*value	0.198	0.379	<0.001	0.694	0.086	0.073	0.817	0.574	0.345^*^	0.207^*^	0.235^*^	0.474^*^
**Occupation**											
Professional and technical	0.44 ± 0.52	0.59 ± 0.54	0.98 ± 0.54	0.27 ± 0.41	0.19 ± 0.40	0.52 ± 0.39	0.20 ± 0.31	0.25 ± 0.30	0.50 ± 0.40	0.07 ± 0.16	0.06 ± 0.21	0.24 ± 0.21
personnel											
Administrative staff	0.41 ± 0.46	0.61 ± 0.59	0.93 ± 0.50	0.21 ± 0.31	0.16 ± 0.40	0.48 ± 0.35	0.18 ± 0.31	0.24 ± 0.34	0.49 ± 0.38	0.07 ± 0.16	0.05 ± 0.16	0.22 ± 0.21
Workers/service staff	0.44 ± 0.54	0.65 ± 0.59	0.88 ± 0.55	0.24 ± 0.43	0.20 ± 0.47	0.50 ± 0.44	0.22 ± 0.32	0.22 ± 0.30	0.49 ± 0.42	0.08 ± 0.22	0.03 ± 0.12	0.23 ± 0.21
Other	0.32 ± 0.46	0.48 ± 0.52	0.82 ± 0.52	0.15 ± 0.26	0.10 ± 0.24	0.39 ± 0.33	0.21 ± 0.38	0.26 ± 0.44	0.48 ± 0.46	0.11 ± 0.27	0.03 ± 0.15	0.24 ± 0.28
Unemployed	0.43 ± 0.55	0.63 ± 0.64	0.85 ± 0.59	0.25 ± 0.44	0.10 ± 0.27	0.48 ± 0.44	0.23 ± 0.39	0.30 ± 0.45	0.56 ± 0.51	0.11 ± 0.24	0.04 ± 0.18	0.27 ± 0.31
*P-*value	0.358	0.206	0.165	0.205^*^	0.051^*^	0.152	0.810	0.730^*^	0.665^*^	0.429^*^	0.626^*^	0.721^*^
**Residence**, ***n*** **(%)**											
City	0.40 ± 0.51	0.62 ± 0.56	0.99 ± 0.51	0.20 ± 0.35	0.19 ± 0.47	0.50 ± 0.39	0.22 ± 0.35	0.27 ± 0.40	0.49 ± 0.42	0.08 ± 0.20	0.04 ± 0.15	0.24 ± 0.23
Town	0.45 ± 0.42	0.62 ± 0.52	0.85 ± 0.51	0.24 ± 0.31	0.15 ± 0.32	0.48 ± 0.32	0.20 ± 0.32	0.21 ± 0.29	0.49 ± 0.39	0.08 ± 0.16	0.04 ± 0.13	0.22 ± 0.20
Rural	0.40 ± 0.55	0.57 ± 0.62	0.83 ± 0.58	0.24 ± 0.43	0.11 ± 0.27	0.46 ± 0.43	0.21 ± 0.36	0.53 ± 0.48	0.53 ± 0.48	0.11 ± 0.24	0.05 ± 0.18	0.25 ± 0.28
*P-*value	0.712	0.596	0.008	0.598	0.197^*^	0.535	0.750	0.498^*^	0.845^*^	0.594^*^	0.789	0.822^*^
**Family monthly income level (yuan)**											
<2,500	0.41 ± 0.57	0.63 ± 0.61	0.74 ± 0.58	0.24 ± 0.42	0.13 ± 0.26	0.45 ± 0.42	0.24 ± 0.41	0.26 ± 0.38	0.50 ± 0.50	0.13 ± 0.30	0.05 ± 0.22	0.26 ± 0.32
2,055–4,999	0.39 ± 0.51	0.57 ± 0.6	0.85 ± 0.55	0.21 ± 0.39	0.11 ± 0.31	0.45 ± 0.40	0.21 ± 0.35	0.25 ± 0.42	0.49 ± 0.45	0.10 ± 0.22	0.04 ± 0.17	0.24 ± 0.27
5,000–9,999	0.47 ± 0.38	0.66 ± 0.6	0.94 ± 0.57	0.28 ± 0.43	0.20 ± 0.47	0.53 ± 0.45	0.20 ± 0.33	0.26 ± 0.37	0.54 ± 0.43	0.06 ± 0.15	0.03 ± 0.12	0.24 ± 0.22
≥10,000	0.33 ± 0.4	0.50 ± 0.48	1.00 ± 0.48	0.18 ± 0.25	0.12 ± 0.24	0.45 ± 0.27	0.21 ± 0.33	0.27 ± 0.36	0.52 ± 0.45	0.10 ± 0.23	0.06 ± 0.18	0.25 ± 0.24
*P-*value	0.170	0.170	0.003	0.200^*^	0.126^*^	0.064^*^	0.639	0.970	0.430^*^	0.096	0.124^*^	0.598^*^
**Number of family children**											
0	0.41 ± 0.51	0.59 ± 0.59	0.87 ± 0.55	0.22 ± 0.39	0.13 ± 0.33	0.47 ± 0.40	0.20 ± 0.34	0.25 ± 0.38	0.51 ± 0.44	0.09 ± 0.21	0.04 ± 0.17	0.24 ± 0.25
≥1	0.43 ± 0.53	0.62 ± 0.55	0.95 ± 0.57	0.28 ± 0.39	0.18 ± 0.44	0.52 ± 0.40	0.30 ± 0.44	0.34 ± 0.43	0.55 ± 0.50	0.14 ± 0.31	0.06 ± 0.17	0.30 ± 0.31
*P-*value	0.792	0.758	0.330	0.292	0.968^**^	0.418	0.095^**^	0.072^*^	0.620^**^	0.072^*^	0.151	0.080^**^
**Phases of ART treatment**											
Before IVF treatment	0.35 ± 0.47	0.56 ± 0.52	0.93 ± 0.53	0.22 ± 0.34	0.12 ± 0.26	0.46 ± 0.35	0.20 ± 0.35	0.25 ± 0.42	0.49 ± 0.46	0.09 ± 0.22	0.05 ± 0.21	0.23 ± 0.27
First cycle of IVF	0.36 ± 0.49	0.55 ± 0.57	0.83 ± 0.51	0.17 ± 0.32	0.12 ± 0.34	0.42 ± 0.36	0.23 ± 0.37	0.26 ± 0.35	0.53 ± 0.44	0.10 ± 0.23	0.04 ± 0.14	0.25 ± 0.26
Second cycle of IVF	0.43 ± 0.55	0.61 ± 0.61	0.85 ± 0.56	0.25 ± 0.42	0.15 ± 0.36	0.48 ± 0.43	0.21 ± 0.31	0.33 ± 0.41	0.57 ± 0.48	0.10 ± 0.21	0.03 ± 0.12	0.27 ± 0.25
Third and later cycle of IVF	0.50 ± 0.53	0.67 ± 0.63	0.91 ± 0.60	0.27 ± 0.44	0.17 ± 0.42	0.53 ± 0.44	0.17 ± 0.30	0.24 ± 0.32	0.44 ± 0.36	0.08 ± 0.24	0.05 ± 0.15	0.21 ± 0.21
*P-*value	0.065	0.354	0.346	0.280	0.565	0.385	0.679	0.227	0.148	0.833	0.761	0.359

* *Kruskal-Wallis test*.

** *Wilcoxon rank test*.

*IVF, in vitro fertilization*.

### Change in the Psychological State and Subgroup Analysis

**Figure 1:**
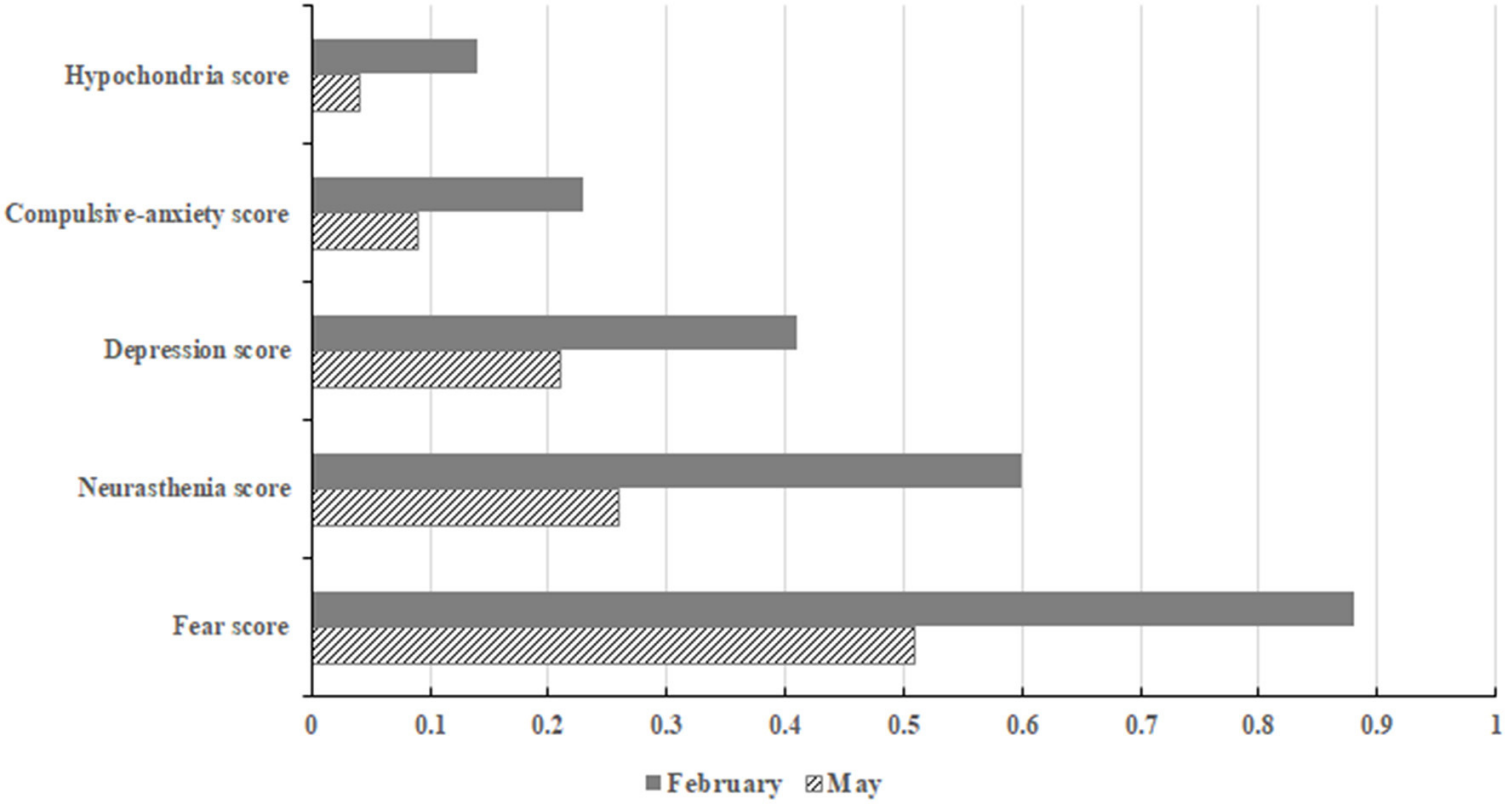
Comparison of psychological scores between ART-treated women during the outbreak period and those during the control period.

Table 3 and Figure 1 illustrate that the ART-treated women's scores for all psychological dimensions during the control period showed a decline compared to the outbreak period with the most significant decline in the scores for the fear dimension (adjusted difference = −0.37; 95% CI = −0.43, −0.31), followed by those for the neurasthenia dimension (difference = −0.31; 95% CI: −0.36, −0.25), the depression dimension (difference = −0.18; 95% CI: −0.23, −0.13), the compulsive-anxiety dimension (difference = −0.13; 95% CI: −0.16, −0.09), and the hypochondria dimension (difference = −0.09; 95% CI: −0.12, −0.06).

**Table 3:** Changes in psychological questionnaire scores between the outbreak period and the control period.

**Variables**	**Outbreak period**	**Control period**	**Difference (95% CI), *P*-value**	Adjusted difference (95% CI)^*^, *P*-value
Depression score	0.41 ± 0.52	0.21 ± 0.35	−0.20 (−0.24, −0.15), <0.001	−0.18 (−0.23, −0.13), <0.001
Neurasthenia score	0.60 ± 0.59	0.26 ± 0.39	−0.33 (−0.38, −0.28), <0.001	−0.31 (−0.36, −0.25), <0.001
Fear score	0.88 ± 0.55	0.51 ± 0.45	−0.37 (−0.42, −0.32), <0.001	−0.37 (−0.43, −0.31), <0.001
Compulsive-anxiety score	0.23 ± 0.39	0.09 ± 0.22	−0.14 (−0.17, −0.11), <0.001	−0.13 (−0.16, −0.09), <0.001
Hypochondria score	0.14 ± 0.34	0.04 ± 0.17	−0.10 (−0.12, −0.07), <0.001	−0.09 (−0.12, −0.06), <0.001
Total score	0.47 ± 0.40	0.24 ± 0.26	−0.23 (−0.26, −0.20), <0.001	−0.22 (−0.25, −0.18), <0.001

* *Adjusted for women's age, education level, occupation, residence location, family monthly income level, number of children in the family, and phase of ART treatment*.

Following subgroup analysis of the change in the psychological dimension scores from the first to the second wave, we found that the scores for all psychological dimensions during the control period decreased among all subgroups, and the differences in the scores were statistically significant (see Supplementary Table 1 for details).

## Discussion

This study investigated the mental health status of ART-treated women during the COVID-19 pandemic for the first time. It assessed the changes in their mental health after the pandemic stabilized in China. A poor psychological state is more likely to occur under the ART process's interruption during the COVID-19 pandemic. Our study found that the mental health of women undergoing ART treatment was mainly manifested as fear during the COVID-19 pandemic in Xi'an, and as the pandemic was brought under control, the mental health of ART-treated women improved.

Following the onset of the pandemic, several studies on pregnant women's mental health reported that the main manifestations were anxiety and depression (23–25). In our study, ART-treated women's psychological state was primarily fearful. After the Zika virus began to spread, Yang observed the mental health of women of childbearing age in the United States and found that women who were pregnant or planning to become pregnant showed a higher sense of fear than women who did not meet these two conditions (26). After the COVID-19 pandemic began in Italy, pregnant women's fear was as high as 49%. Pregnant women who had psychological disorders before the pandemic experienced more severe levels of fear (27). A study in Japan found that the COVID-19 pandemic has had a significant impact on the economy and residents' happiness. High levels of fear and panic behavior were observed in the general population as was a phenomenon of hoarding numerous living materials (28). People's fear and anxiety were often aggravated under the raging pandemic (29, 30). Ahorsu et al. used the Fear of COVID-19 Scale (FCV-19S) to assess pregnant women' fear of COVID-19 and analyze the associations among fear of COVID-19, mental health, and preventive behaviors. They found that fear of COVID-19 among pregnant women was significantly and positively associated with their psychological problems and their preventive COVID-19 behaviors. The fear of COVID-19 among pregnant women was significantly and negatively associated with their mental quality of life (31). In our study, the majority of women treated with ART had begun their first IVF cycle. According to Xi'an's pandemic prevention policies, all non-emergency medical treatments were restricted at the Northwest Women's and Children's Hospital in February 2020, including ART treatment. The restriction of ART treatment during the COVID-19 pandemic led to fear of the decreased odds of conception. Therefore, it was necessary to increase healthcare professionals' sensitivity to ART-treated women's psychological fears and adopt tailor-made intervention measures to provide appropriate psychological support in unusual times.

This study found that ART-treated women's mental health during the control period improved to varying degrees, and their fear and neurasthenia were significantly relieved. As of the control period, the domestic pandemic was in a stable state, and most areas of China resumed work, schooling, and production. Additionally, ART treatment and other non-emergency medical treatment were re-initiated at the Northwest Women's and Children's Hospital in Xi'an in May 2020. The strain on people's mental health has thus been considerably eased. Research by Wu found that pregnant women under the age of 35 were at increased risk of depression and anxiety symptoms during the outbreak of the COVID-19 (25). Guo's study found that the negative impact of the COVID-19 pandemic on livelihoods was particularly severe in regard to mental health problems (β = 0.15; 95% CI: 0.10, 0.19) (32). Therefore, after the pandemic stabilized, the city's blockade was lifted, full-time and middle-income workers resumed their normal life rhythm, economic pressure was relieved, and people's mental state improved.

Epidemiological and zoological studies have also shown that psychological stress was considered a potential cofactor in the pathogenesis of infectious diseases, which can change animals' and humans' sensitivity to sources of infection, thereby affecting the onset, process, and pathology results of certain infectious diseases (33). Therefore, special attention should be paid to the mental health level of populations undergoing ART treatment. Most reproductive societies recommend that, except in a small number of cases, the current cycle of embryo transfer should be postponed, and a new IVF cycle should not be initiated (34). The severity of the COVID-19 outbreak could indirectly impact negative emotions by affecting sleep quality; maintaining an appropriate amount of daily physical exercise and adequate sleep may improve mental health (35). In countries or regions with severe pandemic, remote health care could be implemented and tailored for high-risk prenatal patients (36). Maternal and infant health care institutions should understand pregnant women's needs, optimize prenatal care services, and provide targeted and easily accessible health education and services to ensure mothers and infants (37).

In our study, there is no non-pandemic control group because of the sudden outbreak of the COVID-19. The same questionnaire, the Psychological Questionnaire for Public Health Emergencies, was used in a study on the status of mental health in college students during the SARS pandemic period. Their study included 1,019 college students, 702 of whom were boys, with a mean age of 20.63. Their scores for the depression, neurasthenia, fear, compulsive anxiety, and hypochondria dimensions were 0.33, 0.37, 0.64, 0.24, and 0.20, respectively (22). Compared to that study, the participants in this study were older, and the psychological dimension scores were higher during the outbreak period.

This study has several limitations. First, this is a repeated cross-sectional study rather than a cohort study. A longitudinal cohort study could more accurately assess changes in mental health. Second, although we used multivariable regression to control for potential confounders, the findings may be confounded by unmeasured covariates. Additionally, the interpretation of this study's results may be restricted by time, geography, and socio-cultural background. Finally, instruments designed specifically for COVID-19, such as the Fear of COVID-19 Scale (FCV-19S), were not used in our study, which may affect the accuracy of the psychological assessment in relation to COVID-19. These limitations should be considered when comparing our results with other relevant research results.

## Conclusion

This study found that ART-treated women's mental health during the COVID-19 pandemic was mainly manifested as fear. With the effective control of the COVID-19 pandemic in China, the mental health of ART-treated women improved in all dimensions. The COVID-19 pandemic undoubtedly influences the mental health of women undergoing ART treatment, and clinicians should be aware of this for similar situations in the future.

## Data Availability Statement

The raw data supporting the conclusions of this article will be made available by the authors, without undue reservation.

## Ethics Statement

The studies involving human participants were reviewed and approved by the Human Research Ethics Committee of the Northwest Women's and Children's Hospital (No. 2020001). The patients/participants provided their written informed consent to participate in this study.

## Author Contributions

PQ, MW, WS, and JS conceived and designed the study. PQ, DZ, PJ, SD, WS, MW, and JS drafted and revised the manuscript. PQ and DZ analyzed and interpreted the data. PQ, DZ, and MW collected and cleared the data. All authors have read and approved the final version of the manuscript.

## Conflict of Interest

The authors declare that the research was conducted in the absence of any commercial or financial relationships that could be construed as a potential conflict of interest.
