# Supplementary material for: Changes in Mental Health of Women Undergoing Assisted Reproductive Technology Treatment During the COVID-19 Pandemic Outbreak in Xi'an, China
Source: Front Public Health. 2021 May 25;9:645421. doi: 10.3389/fpubh.2021.645421 (PMC8185191; doi:10.3389/fpubh.2021.645421)
Supplement: Supplementary file 1 [file Table_1.DOCX]

**Supplementary Table 1.** Subgroup analysis of changes in psychological questionnaire scores between the outbreak period and the control period

| Variables | Depression score difference | Neurasthenia  score difference | Fear  score difference | Compulsive-anxiety  score difference | Hypochondria  score difference | Total score difference |
| --- | --- | --- | --- | --- | --- | --- |
| Women’s age (year), n (%) |  |  |  |  |  |  |
| <30 | –0.15 (–0.23, –0.07) | –0.32 (–0.41, –0.23) | –0.33 (–0.43, –0.24) | –0.11 (–0.16, –0.05) | –0.09 (–0.13, –0.05) | –0.20 (–0.26, –0.14) |
| 30–34.99 | –0.23 (–0.30, –0.17) | –0.35 (–0.42, –0.28) | –0.38 (–0.46, –0.31) | –0.15 (–0.19, –0.11) | –0.11 (–0.15, –0.07) | –0.25 (–0.30, –0.20) |
| ≥35 | –0.18 (–0.28, –0.07) | –0.31 (–0.42, –0.20) | –0.39 (–0.50, –0.28) | –0.14 (–0.21, –0.07) | –0.07 (–0.13, –0.02) | –0.22 (–0.30, –0.15) |
| Education level, n (%) |  |  |  |  |  |  |
| Junior high school and below | –0.20 (–0.30, –0.10) | –0.33 (–0.44, –0.22) | –0.24 (–0.36, –0.12) | –0.13 (–0.20, –0.05) | –0.05 (–0.09, –0.00) | –0.20 (–0.28, –0.12) |
| High school/secondary school | –0.12 (–0.23, –0.02) | –0.28 (–0.39, –0.17) | –0.27 (–0.39, –0.14) | –0.13 (–0.21, –0.05) | –0.06 (–0.12, –0.01) | –0.17 (–0.25, –0.09) |
| College | –0.16 (–0.25, –0.08) | –0.35 (–0.45, –0.25) | –0.41 (–0.51, –0.32) | –0.11 (–0.16, –0.05) | –0.10 (–0.15, –0.05) | –0.22 (–0.28, –0.16) |
| University and above | –0.26 (–0.34, –0.19) | –0.35 (–0.44, –0.27) | –0.47 (–0.55, –0.39) | –0.17 (–0.22, –0.12) | –0.10 (–0.12, –0.07) | –0.29 (–0.34, –0.24) |
| Occupation, n (%) |  |  |  |  |  |  |
| Professional and technical personnel | –0.24 (–0.34, –0.14) | –0.34 (–0.45, –0.24) | –0.48 (–0.59, –0.36) | –0.20 (–0.27, –0.12) | –0.13 (–0.21, –0.06) | –0.28 (–0.36, –0.21) |
| Administrative staff | –0.22 (–0.33, –0.11) | –0.37 (–0.50, –0.24) | –0.43 (–0.56, –0.31) | –0.14 (–0.21, –0.08) | –0.11 (–0.19, –0.03) | –0.26 (–0.34, –0.18) |
| Workers/service staff | –0.22 (–0.33, –0.11) | –0.43 (–0.54, –0.32) | –0.39 (–0.51, –0.26) | –0.16 (–0.24, –0.08) | –0.16 (–0.24, –0.09) | –0.27 (–0.36, –0.19) |
| Other | –0.11 (–0.21, –0.02) | –0.22 (–0.33, –0.11) | –0.33 (–0.44, –0.22) | –0.04 (–0.10, 0.02) | –0.06 (–0.11, –0.02) | –0.15 (–0.22, –0.08) |
| Unemployed | –0.20 (–0.28, –0.11) | –0.33 (–0.43, –0.23) | –0.29 (–0.39, –0.19) | –0.14 (–0.20, –0.08) | –0.06 (–0.10, –0.02) | –0.21 (–0.28, –0.14) |
| Residence, n (%) |  |  |  |  |  |  |
| City | –0.18 (–0.26, –0.09) | –0.35 (–0.44, –0.26) | –0.50 (–0.59, –0.41) | –0.13 (–0.18, –0.07) | –0.15 (–0.22, –0.09) | –0.26 (–0.32, –0.20) |
| Town | –0.25 (–0.34, –0.15) | –0.41 (–0.51, –0.31) | –0.36 (–0.48, –0.25) | –0.17 (–0.23, –0.11) | –0.11 (–0.17, –0.05) | –0.26 (–0.33, –0.19) |
| Rural | –0.19 (–0.25, –0.13) | –0.30 (–0.37, –0.24) | –0.31 (–0.38, –0.23) | –0.13 (–0.18, –0.09) | –0.06 (–0.09, –0.03) | –0.21 (–0.25, –0.16) |
| Family monthly income level (yuan), n (%) |  |  |  |  |  |  |
| <2500 | –0.16 (–0.29, –0.04) | –0.37 (–0.50, –0.25) | –0.24 (–0.37, –0.10) | –0.11 (–0.20, –0.02) | –0.08 (–0.14, –0.02) | –0.19 (–0.28, –0.10) |
| 2055–4999 | –0.18 (–0.25, –0.11) | –0.32 (–0.40, –0.23) | –0.36 (–0.44, –0.27) | –0.11 (–0.16, –0.06) | –0.07 (–0.11, –0.03) | –0.21 (–0.26, –0.15) |
| 5000–9999 | –0.28 (–0.36, –0.19) | –0.39 (–0.48, –0.30) | –0.40 (–0.50, –0.31) | –0.22 (–0.28, –0.16) | –0.17 (–0.23, –0.11) | –0.30 (–0.36, –0.23) |
| ≥10000 | –0.13 (–0.21, –0.04 | –0.23 (–0.33, –0.13) | –0.48 (–0.59, –0.37) | –0.08 (–0.14, –0.02) | –0.06 (–0.11, –0.01) | –0.20 (–0.27, –0.14) |
| Number of family children |  |  |  |  |  |  |
| 0 | -0.21 (-0.25, -0.16) | -0.34 (-0.39, -0.29) | -0.37 (-0.42, -0.31) | -0.14 (-0.17, -0.10) | -0.10 (-0.12, -0.07) | -0.23 (-0.27, -0.20) |
| ≥1 | -0.12 (-0.28, 0.04) | -0.28 (-0.44, -0.12) | -0.40 (-0.58, -0.23) | -0.14 (-0.26, -0.03) | -0.12 (-0.22, -0.02) | -0.21 (-0.33, -0.10) |
| Phases of ART treatment |  |  |  |  |  |  |
| Before IVF treatment | -0.15 (-0.22, -0.07) | -0.31 (-0.40, -0.23) | -0.44 (-0.53, -0.35) | -0.13 (-0.18, -0.08) | -0.07 (-0.11, -0.03) | -0.23 (-0.28, -0.17) |
| First cycle of IVF | -0.13 (-0.22, -0.04) | -0.29 (-0.38, -0.20) | -0.30 (-0.40, -0.19) | -0.07 (-0.13, -0.02) | 0.08 (-0.13, -0.04) | -0.17 (-0.24, -0.11) |
| Second cycle of IVF | -0.21 (-0.32, -0.11) | -0.28 (-0.40, -0.16) | -0.28 (-0.40, -0.17) | 0.14 (-0.22, -0.06) | -0.12 (-0.18, -0.05) | -0.21 (-0.29, -0.13) |
| Third and later cycle of IVF | -0.33 (-0.47, -0.19) | -0.43 (-0.60, -0.27) | -0.48 (-0.64, -0.31) | -0.19 (-0.31, -0.07) | -0.12 (-0.23, -0.01) | -0.32 (-0.43, -0.20) |
